# Toward neural health measurements for cochlear implantation: The relationship among electrode positioning, the electrically evoked action potential, impedances and behavioral stimulation levels

**DOI:** 10.3389/fneur.2023.1093265

**Published:** 2023-02-09

**Authors:** Lars Lambriks, Marc van Hoof, Joke Debruyne, Miranda Janssen, Janny Hof, Katja Hellingman, Elke Devocht, Erwin George

**Affiliations:** ^1^Department of ENT/Audiology, School for Mental Health and NeuroScience, Maastricht University Medical Centre, Maastricht, Netherlands; ^2^Department of Methodology and Statistics, Care and Public Health Research Institute, Maastricht University, Maastricht, Netherlands

**Keywords:** cochlear implant, ECAP, electrode positioning, impedances, stimulation levels, neural health

## Abstract

**Introduction:**

Estimating differences in neural health across different sites within the individual cochlea potentially enables clinical applications for subjects with a cochlear implant. The electrically evoked compound action potential (ECAP) is a measure of neural excitability that possibly provides an indication of a neural condition. There are many factors, however, that affect this measure and increase the uncertainty of its interpretation. To better characterize the ECAP response, its relationship with electrode positioning, impedances, and behavioral stimulation levels was explored.

**Methods:**

A total of 14 adult subjects implanted with an Advanced Bionics cochlear electrode array were prospectively followed up from surgery to 6 months postoperative. Insertion depth, distance to the modiolus, and distance to the medial wall were assessed for each electrode by postoperative CT analysis. ECAPs were measured intraoperatively and at three visits postoperatively on all 16 electrodes using the NRI feature of clinical programming software and characterized using multiple parameters. Impedances and behavioral stimulation levels were measured at every fitting session.

**Results:**

Patterns in ECAPs and impedances were consistent over time, but high variability existed among subjects and between different positions in the cochlea. Electrodes located closer to the apex of the cochlea and closer to the modiolus generally showed higher neural excitation and higher impedances. Maximum loudness comfort levels were correlated strongly with the level of current needed to elicit a response of 100 μV ECAP.

**Conclusion:**

Multiple factors contribute to the ECAP response in subjects with a cochlear implant. Further research might address whether the ECAP parameters used in this study will benefit clinical electrode fitting or the assessment of auditory neuron integrity.

## 1. Introduction

Cochlear implantation is the main treatment strategy for patients with severe to profound sensorineural hearing loss. Although most cochlear implant (CI) users receive a substantial benefit in speech recognition (1), performance varies highly among recipients (2, 3). This variability may be partially explained by the individual electrode–neuron interface, which refers to electrode positioning and auditory neuron integrity (4–6). Some areas of the cochlea are potentially less viable to be stimulated electrically due to the occurrence of retracting neurites and reduced integrity of spiral ganglion cells and dead regions, a common phenomenon in sensorineural hearing loss (7). The electrode array, however, is surgically inserted without any knowledge of these differences in neural health within the cochlea. Therefore, some contacts are located at cochlear sites, where successful transmission of information is not possible. Estimating individual differences in neural health potentially enables clinical applications such as focused stimulation or adapting frequency assignment of electrodes based on healthy neural regions (8).

Since there are no current techniques available to directly measure neural health *in vivo*, researchers have searched for derivative measures that might provide an indication of the neural condition at individual sites along the cochlea. One of these measures is the electrically evoked compound action potential (ECAP), which represents the summed response of a group of electrically activated spiral ganglion cells in the cochlea. Despite the clinical tools available to measure ECAPs, there are no methods to implement ECAP measurements in clinical fitting to improve CI outcomes yet. Partly, this might be due to uncertainties in the interpretation of ECAP responses. To evaluate ECAPs as a measure of neural health, it may need to be interpreted in relation to multiple factors such as the distance between neurons and electrodes, impedances of surrounding tissue, current paths, and current spread (9). In the current study, the relationship between ECAP responses and electrode location, impedances, and behavioral stimulation levels will be explored. Differences between patients will be assessed, and different derivatives of the ECAP response are investigated with the goal of better characterizing neural health in the future.

Electrically evoked compound action potential responses can be measured using the telemetry function of clinical audiological software. Neural response imaging (NRI) records electrical activity on one electrode while stimulating an electrode close to the recording contact. After artifact removal, the resulting ECAP response typically shows a triphasic waveform, with an initial positive peak (P1) followed by a negative trough (N1) followed by a second positive peak (P2) (10). The amplitude of the ECAP response is hereby defined as the absolute difference between N1 and P2. By aligning the amplitude of ECAP responses as a function of stimulation level, an ECAP growth function is created. Typically, ECAP growth functions are used to derive thresholds (minimum stimulation level needed for a detectable ECAP response), slopes (speed of the increase in growth), or amplitudes at specified stimulation levels.

Currently, the main clinical applications of ECAP are to provide information on implant function over time and to provide estimations of fitting levels in patients without reliable behavioral responses (10, 11). Since ECAP is generated by the activation of surviving auditory neurons, it may also be a potential tool to estimate neural health. In animals, several features of the ECAP response have been related to histological data. Amplitude and slope of the ECAP growth function are correlated with spiral ganglion cell density in deafened guinea pigs (12, 13). Moreover, Prado-Guitierrez et al. (13) demonstrated a correlation between auditory neural survival and ECAP variations in pulse duration and interphase gap. In humans, a direct comparison between ECAP and ganglion cell count is not feasible *in vivo*. Studies have linked characteristics of ECAP measures to speech perception results, but surprisingly found inconsistent results (6, 14–18).

An important confounding aspect of the relationship between ECAP measures and neural health is that neural response will be influenced by the position within the cochlea. For example, Van de Heyning et al. (19) reported greater ECAP amplitudes at various stimulation levels and steeper slopes of the ECAP growth function for electrodes located apically in the cochlea compared to contacts located in the basal region. Other studies have also found that apical contacts generate greater ECAP amplitudes (20, 21), which is potentially explained by better neural survival and a smaller distance to the medial wall toward the apex. Indeed, Degen et al. (22) and Schvartz-Leyzac et al. (9) found more elevated ECAP thresholds for electrodes located further from the modiolus. Given these results, the question remains whether the ECAP response can be interpreted as a direct representation of neural health or should be corrected for the position of the electrode in the cochlea to be interpreted as such. In addition, studies have explored the relationship between ECAP measures and behavioral stimulation levels. Clinical programming of a CI processor requires the fitting of the maximum comfortable level and the lowest level that patients can hear (M level/T level, terminology varies among manufacturers). Overall, studies only show a moderate correlation between ECAP thresholds and M- and T levels (11, 23). This lack of a clear relationship is possibly affected by the difference between stimuli presented in ECAP measures and behavioral procedures (16, 24).

Impedance telemetry is clinically applied to check device integrity during the whole lifespan of CI use by detecting abnormalities such as open and short circuits. Electrode impedance reflects the amount of resistance to the flow of electrical current from a stimulating electrode to a receiving electrode. Therefore, it might indicate variations in the tissues surrounding the electrode and its resistive properties. Some studies have reported higher impedances for apical contacts than basal contacts (25, 26), while others found an inverse relationship (27). No clear relationship is found between impedances and distance to the modiolus (28). Zarowski et al. (29) reported significant correlations between impedances and behavioral stimulation levels, but other studies did not confirm this relationship (30, 31).

In the present study, multiple CI variables related to the electrode–neuron interface were measured longitudinally. The main goal was to identify whether electrode location (insertion depth, modiolar proximity, and distance to the medial wall) affected ECAP response. Specifically, it was hypothesized that electrodes located toward the apex and closer to the modiolus/medial wall would elicit higher neural responses. In addition, relationships between electrode position and both impedances and behavioral fitting levels were explored. It also investigated whether ECAP responses were related to impedances and behavioral stimulation levels. To evaluate electrode location within this patient group, an imaging analysis method was used which was previously implemented in a case report study (32). A subgoal of the current study included evaluating the feasibility of this method using a larger sample size. Finally, changes in ECAP, impedances, and behavioral stimulation levels within the first 6 months of CI rehabilitation were investigated.

## 2. Materials and methods

### 2.1. Ethical approval

This study has been approved by the ethics committee of the Maastricht University Medical Center (MUMC+), registered in the Clinical Trials Register (NL64874.068.18), and conducted in accordance with the Declaration of Helsinki. Subjects provided informed consent before participation and were compensated for their traveling costs.

### 2.2. Subjects

Fourteen adult Dutch-speaking patients participated in this study (11 men and three women, mean age: 67 years, SD: 7 years). A diverse range of etiologic factors for hearing loss was present within this patient group (Table 1). All participants received a unilateral HIRes Ultra implant with a HiFocus Midscala electrode of the brand Advanced Bionics^TM^ (Valencia, United States) in the MUMC+. The insertion length of the Midscala electrode is 18.5 mm with 16 single-channel medial electrode contacts spaced at 1 mm intervals. Implantation took place during routine cochlear implant surgery using a round window approach and without dexamethasone or prednisone administration. Here, the electrode array was inserted with a free-hand approach using a stylet. Access to the round window was gained *via* mastoidectomy and posterior tympanotomy. In general, the tip of the array was inserted into the cochlea up to the distal marker at the apical end. Then, the stylet was fixed while pushing the array slowly off the stylet until the proximal blue marker was reached, as specified in the surgical manual. Additional subject characteristics are shown in Table 1.

**Table 1:** Subject characteristics.

							**Etiology (bilateral)**	
**Subject**	**Gender**	**Implanted side**	**Age at implantation (years)**	**Duration of hearing loss (years)**	**Onset hearing loss**	**Type of loss**	**Course of loss**	**Cause of loss**
EP01	M	R	64	15	AO	SN	Progressive	Unknown
EP02	M	R	67	12	AO	SN	Episodic	Unknown
EP03	M	L	62	14	AO	SN	Episodic	COM
EP04	F	L	61	30	AO	SN	Progressive	Suspected autoimmune
EP05	M	R	78	29	JO	SN	Episodic	COM/LL after bilateral RM
EP06	M	L	54	24	AO	SN	Progressive	Unknown
EP07	M	R	62	26	AO	SN	Progressive	Unknown
EP08	M	R	78	23	AO	SN	Sudden	Labyrinthitis
EP09	F	R	64	31	AO	SN	Progressive	Unknown
EP10	M	L	78	14	AO	SN	Progressive	Unknown
EP11	F	R	60	11	JO	SN	Progressive	COM
EP12	F	R	71	39	JO	SN	Progressive	COM
EP13	M	R	65	39	AO	SN	Progressive	Suspected hereditary
EP14	M	R	70	31	AO	M	Progressive	Otosclerosis/hereditary

Duration of hearing loss is defined as the number of years from the onset of self-reported hearing aid use in the implanted ear till CI activation. M, male; F, female; R, right; L, left; AO, adult onset; JO, juvenile onset; SN, sensori-neural; M, mixed; COM, chronic otitis media; LL, liquor leakage; RM, radical mastoidectomy.

### 2.3. Design

Subjects were part of a clinical trial in which an imaging-based fitting strategy was implemented as an intervention from the start of their CI rehabilitation (33). Fitting of subjects was performed with a research processor using research software (Bionic Ear Programming System Plus, BEPS+) with real-life adjustments based on behavioral M and T levels, as it is a part of the clinical routine (in Soundwave^TM^). Primary trial outcomes will be published in a separate manuscript. The overall study overview can be consulted in the previous protocol publication (33). Table 2 shows the schedule of measurements of those outcomes that are included in the current manuscript. Subjects were followed up from surgery to 6 months postoperative. ECAP, impedances, and behavioral stimulation levels were measured at multiple visits. All subjects completed the study, except for EP07, who was terminated from the study 3 months after CI activation.

**Table 2:** Schedule of assessments and measurements where timepoint 0 represents CI activation (first fitting).

			**Weeks after CI activation**														
**Outcomes**	**#**	**I**	−**3**	**0**	**1**	**2**	**3**	**4**	**5**	**6**	**7**	**8**	**10**	**12**	**16**	**20**	**26**
Clinical CT/MRI	X																
CBCT scan			X														
Residual hearing	X			X													
ECAP		X			X							X					X
Impedances		X		X	X	X	X	X	X	X	X	X	X	X	X	X	X
Behavioral stimulation levels					X					X							X

The preoperative measurement of the residual hearing was administered at least within 1 year before implantation. ECAP (evoked compound action potential), impedances, and behavioral thresholds were recorded as measures of implant function and were related to electrode positioning. #, preoperative; I, intraoperative; CBCT, cone beam computed tomography.

### 2.4. Imaging

As part of the standard CI-candidacy workup, a CT scan and an MRI scan (if clinically indicated to exclude cochlear abnormalities) were performed for each patient. One week after surgery, a Cone Beam CT (CBCT) scan was performed to assess the surgical placement of the cochlear implant. Pre- and postoperative images (CT and CBCT, or MRI and CBCT when available) were fused (34) using 3D Slicer (35) and BRAINSFit software (36). 3D visualizations of the cochlear labyrinths were created using the volume rendering functionality in the 3D slicer. Intracochlear electrode positioning was assessed by placing markers at the center of each contact (32). Here, electrode 1 is defined as the most apical electrode (lowest tonotopic frequency) and 16 as the most basal contact (highest tonotopic frequency). The lateral wall (LW) was marked from start at the round window to the helicotrema at a height corresponding to the basilar membrane. Here, fiducials were placed manually using three reconstruction planes to follow the lateral wall closely. This resulted in a *post-hoc* calculated mean distance of 0.27 mm between individual markers. Since determining the full trajectory of the medial wall (MW) was not always possible due to insufficient image quality, fiducials were not placed along the full extent of the MW, but only at those locations that were closest to electrode contacts in order to identify the electrode–MW distances. The center of the modiolus was delineated by a line connecting the modiolus at the base and apex of the cochlea. Euclidean distances from electrodes to the LW, MW, and the modiolar axis were calculated. Insertion depth was calculated by first identifying the nearest points on the LW for each contact and then calculating the distance from the round window to these points across the interpolated LW. For each electrode, insertion depth was recorded as the absolute distance from the round window and as the fractional depth relative to the subject's cochlear length. Both insertion depth and cochlear morphology (height and length) were also described as angular parameters, where the 0° angle was defined as the axis from the round window to the modiolar axis. For cochlear morphology, the angle between successive measurements on the lateral wall for the same points and the middle of the modiolus was used to visualize how the cochlea was extending in size and height (vs. the round window). In addition, tonotopic electrode frequency was calculated by applying the original Greenwood function for an average human cochlea to the insertion depth relative to the subject's cochlear duct length (32, 37). As such, this parameter reflects the frequency according to the tonotopic organization of the cochlea in line with the location of the electrode. Also, the occurrence of translocations of the electrode array from the scala tympani to the scala vestibuli was rated with visual inspection by an experienced observer.

### 2.5. Residual hearing

Pure-tone audiometric thresholds were collected unaided using headphones at frequencies from 250 up to 8,000 Hz. If no response could be recorded within the limits of the audiometer, a value of 5 dB HL greater than the maximum tested level was entered. The pure-tone average (PTA) was calculated as the mean of thresholds at 500, 1,000, and 2,000 Hz.

### 2.6. Electrically evoked compound action potential

Electrically evoked compound action potentials were measured on all 16 electrodes using the NRI feature of the clinical programming software of Advanced Bionics (Soundwave). Stimuli were transmitted through a spare Naida Q90 processor (not in use by the patient) which was connected to a clinical programming interface (CPI-3). The initial stimulation level was set to 100 clinical programming units (CU) for intraoperative measurements and 50 CU postoperatively. The stimulation level was increased with manually judged increments (on average 125 CU per step intraoperatively and 50 CU postoperatively). The current was increased to a maximum of 750 CU when necessary for intraoperative measurements while postoperative measurements were limited by the maximum comfortable level of the individual subject. Default stimulus parameters of the Soundwave software were maintained during measurements. The software uses a biphasic pulse pattern with a pulse width of 32 μs and a stimulation rate of 30 pps. The recording electrode was located two electrodes apical from the stimulating electrode, except for the most apical electrode where the recording electrode was two electrodes basal from the stimulating electrode. To reduce stimulus artifacts, the alternating-polarity method was used (10, 15). Here, stimulation is delivered with both a cathodic-leading and anodic-leading pulse. The two measurements are then averaged to remove the stimulation artifact and extract the ECAP signal.

Electrically evoked compound action potential responses are defined as the voltage difference between P2 and N1 peaks. These peaks were recorded as automatically detected by Soundwave but were modified by an experienced observer if considered necessary. ECAP growth functions were constructed by applying second-order interpolation between the minimum and maximum recorded pairs of stimulation level and ECAP response. If missing ECAP values occurred for specific electrodes at the lowest stimulation level, these values were imputed. Three outcome measures were derived out of the ECAP growth functions per stimulating electrode: the 100 μV ECAP, the mean amplitude response ratio, and the interquartile range (IQR) amplitude response ratio. The 100 μV ECAP is described as the first stimulus level that elicits an ECAP response of 100 μV. This response is well-above the NRI noise floor and was expected to be reached in the majority of patients (10, 19). If no eCAP response of 100 μV or higher was reached for a given electrode, that response was registered as missing. The mean amplitude response ratio is described as the mean of ratios between stimulation level and ECAP response. In other words, the ratio was calculated for each pair in the ECAP growth function with the mean overall ratios of the same electrode being recorded as the final outcome. Here, a value of 1 reflects a mean linear relationship between stimulation level and ECAP, while a value higher than 1 indicates increased ECAP responses for an equal increase in stimulation, and a lower than 1 indicates a reduced ECAP response. The IQR amplitude response is calculated over the same ratios but expressed as an interquartile range instead of a mean to parameterize the distribution of values.

### 2.7. Impedance measurements

Impedances were measured during surgery (after ECAP measurement) with the clinical software Soundwave and at the beginning of each fitting session with BEPS+. The impedance measurement was performed in the monopolar (MP) mode. The diagonal values (kOhm) of the impedance matrix were used, whereby the stimulating and recording electrode were the same.

### 2.8. Behavioral stimulation levels

Measurement of behavioral maximum comfortable level (M level) and threshold level (T level) was performed in BEPS+ during fitting sessions. Here, a fixed pulse width of 40.4 μs was used, and the current level was increased while subjects judged loudness perception on a 9-point VAS scale. T levels were noted when stimulus detection occurred (“just audible”). M levels were recorded when perception reached a level between “good” and “loud”. Measurements were performed on five stimulation channels across the array, using electrode combinations 1–2, 4–5, 7–8, 11–12, and 15–16.

### 2.9. Data analysis

Mathematica software 13.0 was used for the analysis and visualization of data. Median and interquartile ranges (IQR) were used for descriptives. Given the small sample size, the likelihood of non-linear relationships, and after visual inspection of the data, Spearman correlations were calculated to assess relationships between electrode positioning and ECAP responses, impedances, and behavioral fitting levels within subjects. An additional analysis was performed to examine Spearman correlations between ECAP responses and both impedances and behavioral stimulation levels. For M and T levels, which are channel (electrode pair) outcomes in contrast to all other electrode-based outcomes, correlations were calculated using the apical electrode of each pair (e.g., the M-level of channel 1–2 was correlated to the ECAP on electrode 1). Analysis was reported for measurements conducted intraoperatively and 1 week after CI activation. These visits had low levels of missing data and concerned two distinct clinical time points (before and after electrical exposure). Bonferroni correction was applied to correct for multiple testing. Here, the alpha level of 0.05 was divided by 30 (0.0017) and rounded to 0.001 to simplify interpretation.

## 3. Results

### 3.1. Cochlear morphology

**Figure 1:**
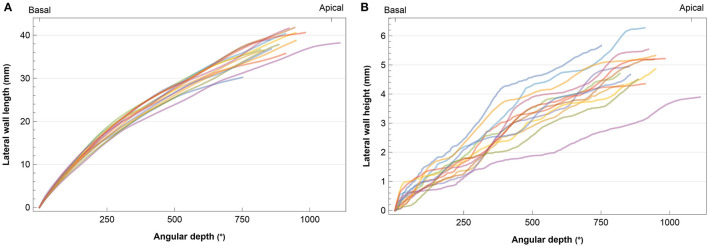
Individual cochlear angular depth in relation to **(A)** length and **(B)** height of the lateral wall. Each subject is represented by a different color.

Figure 1 shows the observed diversity in cochlear length and height based on preoperative CT scans. The length of the LW ranged from 30.21 to 41.76 mm (median 38.48, IQR 3.85). The median height of the LW of the cochlea was estimated at 4.90 mm (range 3.90–6.28, IQR 0.82).

### 3.2. Electrode positioning

#### 3.2.1. Imaging

**Figure 2:**
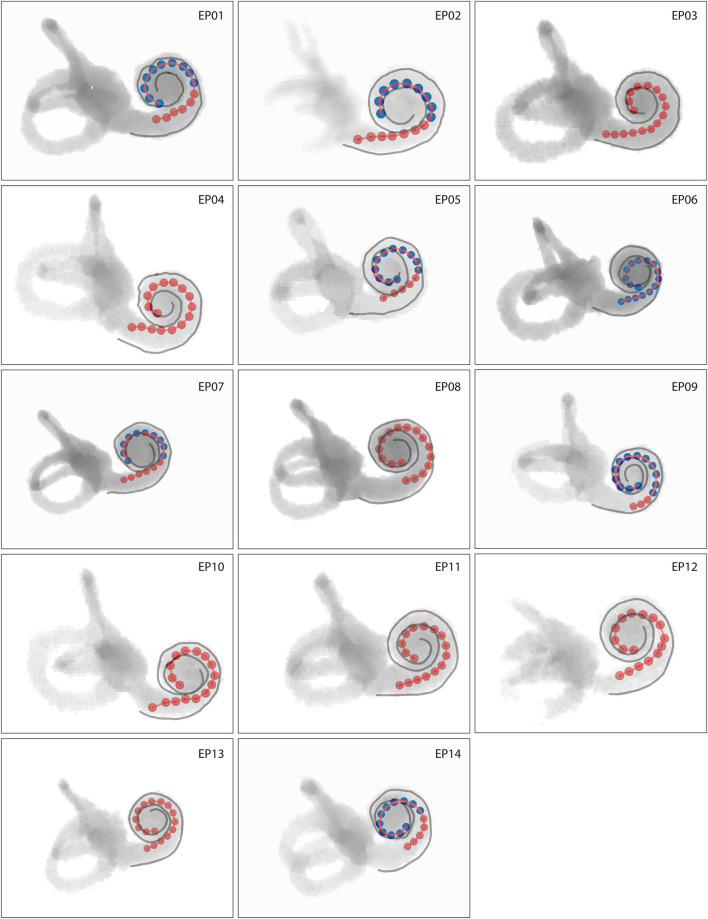
Volume rendering of cochlear labyrinths where red markers represent individual electrodes and gray lines the lateral wall. Intracochlear scalar positions of electrodes are coded by color, with red electrodes located in scala tympani and blue electrodes located in scala vestibuli. Cochleas presented on the same scale show actual interindividual representations in size.

CT volume renderings of cochlear labyrinths in Figure 2 show variability in electrode positioning and cochlear morphology between subjects. The red markers located within the cochlea represent individual electrodes and the LW is highlighted in gray. A high level of variability existed in the insertion depth and curvature (e.g., compare EP02 and EP08) of the same electrode array within the cochlea. All subjects presented a complete insertion with no electrode contacts located outside the cochlea.

#### 3.2.2. Scalar location

In all patients, 64% of all electrode contacts were located in scala tympani. Scalar translocation, in which the electrode array shifts from scala tympani to scala vestibuli, occurred in seven out of 14 subjects (Figure 2). A full scala vestibuli insertion occurred in one subject (EP06). Translocation occurred most often at the cochlear depth of electrodes 10–13, which were located at an angular depth of 115–136° (Supplementary Table 1).

#### 3.2.3. Insertion depth and within-scala positioning

**Figure 3:**
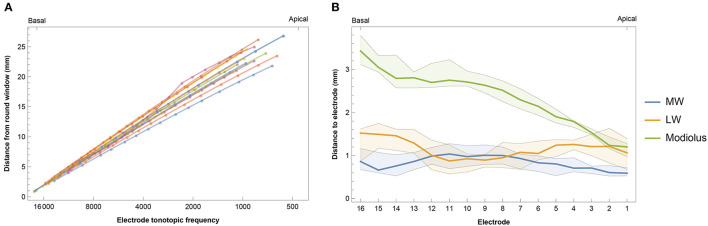
**(A)** Intracochlear insertion depth for 14 subjects in relation to the calculated tonotopic location of each electrode. Insertion depth was calculated as the distance from the round window to the nearest point on the lateral wall for each contact. **(B)** Median intracochlear distances between electrodes and the medial wall (MW), lateral wall (LW), and center of the modiolus. Bands indicate the first and third quartiles.

The insertion depth of the most apical electrode contact ranged from 20.85 to 26.86 mm with a median of 23.73 mm (IQR 2.72 mm). This corresponded to an electrode tonotopic frequency of 566–1,146 Hz with a median of 852 Hz (IQR 318 Hz), as shown in Figure 3A and Supplementary Table 1. Angular insertion depth showed a range of 337–475° with a median of 412° (IQR 47°). In general, the electrode-to-modiolus distance was largest for basal contacts and decreased gradually toward the apex (Figure 3B). From a medial-lateral perspective, Figure 3 shows that the basal portion of the electrode array was often located closer to the medial wall, then leaned more toward the lateral wall for the contacts in the middle of the array, and eventually shifted back toward the medial wall in the apical portion of the cochlea.

### 3.3. Hearing loss after surgery

Subjects had considerable residual hearing in the implanted ear in the low frequencies before surgery (Supplementary Figure 1). Residual hearing deteriorated due to the CI surgical procedure. Median PTA across patients was 94 dB HL (IQR 28 dB HL) pre-surgery and 118 dB HL (IQR 23 dB HL) post-surgery. In those subjects with a scalar translocation (Figure 2), the residual hearing was 105 dB HL (IQR 21) before surgery and 117 dB HL (IQR 29) afterward, compared to 93 dB HL (IQR 36) and 120 dB HL (IQR 23) in subjects without translocation.

### 3.4. Electrically evoked compound action potential

**Figure 4:**
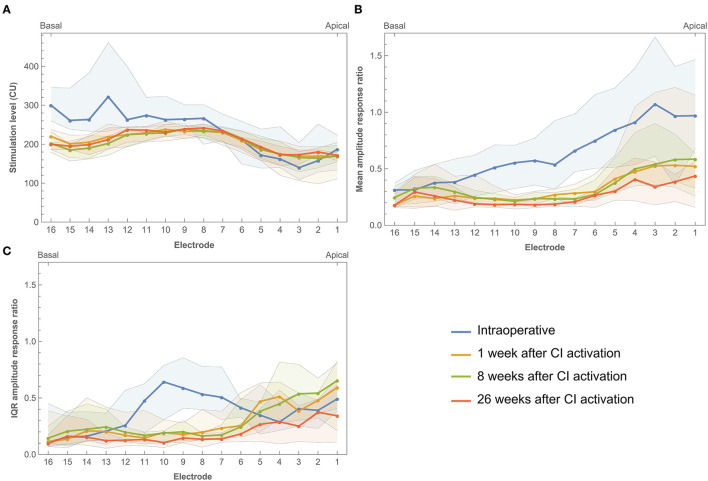
Electrically evoked compound action potential estimates across the electrode array measured intraoperatively and during three return visits within the first 6 months of CI rehabilitation. **(A)** Median 100 μV ECAP, which describes the stimulation level needed to elicit an ECAP response of 100 μV. **(B)** Mean amplitude response ratio, which describes the mean of ratios between stimulation level and ECAP response where a higher value indicates more ECAP response for a given input. **(C)** IQR amplitude response ratio, which describes the interquartile range of ratios between stimulation level and ECAP response. Bands indicate the first and third quartiles.

Electrically evoked compound action potentials were measured during surgery and at three postoperative return visits within the first 6 months of CI rehabilitation. In this study, three estimates of neural response were derived: 100 μV ECAP, mean amplitude response ratio, and IQR amplitude response ratio. In some patients or specific electrodes, 100 μV ECAP was not reached due to the unresponsiveness of neurons and/or the comfortable stimulation level being too low. This was the case in 7.1% of electrodes when measured intraoperatively, and 23.2, 26.1, and 48.1% of electrodes during the three postoperative visits, respectively. These data were recorded as missing and were not included in the analysis. Figure 4A shows that basal and middle electrodes generally required higher stimulation levels to reach an ECAP response of 100 μV ECAP than apical electrodes. Over time, 100 μV ECAP levels remained stable during the first 6 months of CI rehabilitation. Intraoperative measurements showed greater variation at 100 μV ECAP between subjects and less responsiveness at the basal portion of the electrode array compared to postoperative measurements.

Mean amplitude response ratio was higher toward the apical portion of the electrode array, most prominently during surgery (Figure 4B). For example, electrodes 1, 2, and 3 showed a ratio close to 1.0 (with large IQRs between 0.9 and 1.0), which indicates a more linear relationship between input and output, meaning that every increase in stimulation level elicited a similar increase in ECAP response. The mean amplitude response ratio at these electrodes was lower during postoperative visits (for example, between 0.4 and 0.5 at 26 weeks after CI activation). However, ratios at apical electrodes were still higher compared to their basal counterparts. This indicates higher ECAP responses for increasing stimulation inputs toward the apical electrodes. Roughly, a similar pattern was prominent for IQR amplitude response ratio, with the exception of a peak between electrodes 6–11 and the absence of further increase toward the apical electrodes.

**Figure 5:**
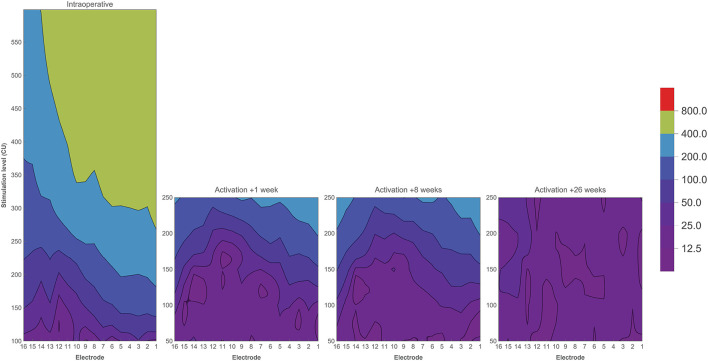
Contour plots of one subject (EP08) showing ECAP responses (μV) in relation to stimulation level (CU) across the electrode array. Colors indicate ECAP values (μV) according to the legend. Measurements have been performed during CI surgery, and at three return visits during the first 6 months of CI rehabilitation.

Complete individual ECAP growth functions for each electrode and across subjects were visualized in contour plots [single patient (EP08) shown in Figure 5, all data in Supplementary Figure 2]. Plotting the full ECAP input–output function enables the evaluation of patterns on an individual level across the array and over time. In general, neural excitation patterns varied individually and were strongly non-linear. Most subjects showed a decrementing slope during surgery, with relatively stable follow-up measurements. Some subjects, such as EP02, EP09, and EP14, showed distinctly different patterns during surgery. Subject EP01, EP03, and EP08 showed a distinct change during follow-up measurements, either in part of the array or on the full array. For example, in subject EP08, stimulation levels between 150 and 250 CU elicited higher ECAP responses at the beginning of CI rehabilitation than after 6 months across the whole array. EP01 showed a similar decrease in ECAP response over time but only for the most apical electrodes.

### 3.5. Impedances

**Figure 6:**
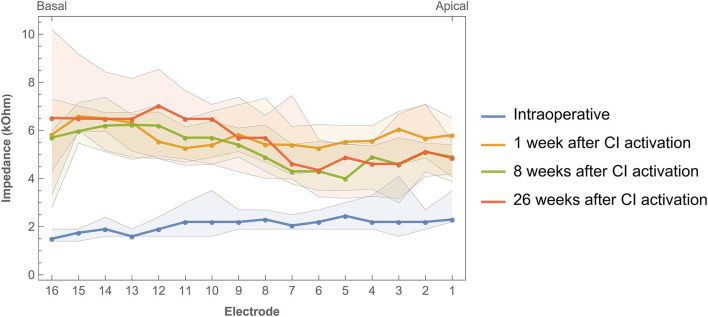
Impedances across the electrode array were measured intraoperatively and during three return visits within the first 6 months of CI rehabilitation. Bands indicate the first and third quartiles.

**Figure 7:**
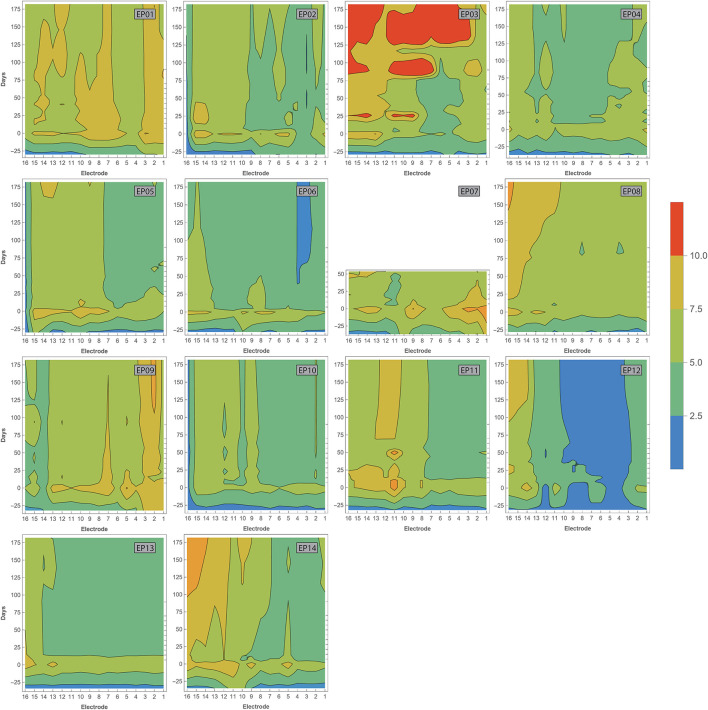
Contour plots showing varying impedances with time (in days, vertical axis) across the electrode array (horizontal axis). Measurements have been performed during surgery, and at every fitting session during the first 6 months of CI rehabilitation. Day 0 represents the first fitting. Colors indicate impedance values according to the legend. Open and short circuits are classified as impedances > 30 and 0.5–1.0 kOhm, respectively.

Impedance values were measured intraoperatively and at every fitting session during the first 6 months of CI rehabilitation. No abnormal open or short circuits occurred throughout the study. Over time, impedances were lower intraoperatively (median 2.07 kΩ, IQR 1.02 kΩ) than 1 week after CI activation (median 5.75 kΩ, IQR 1.92 kΩ). As illustrated in Figure 6, postoperative impedances were higher for basal electrodes compared to apical contacts. As with ECAP, impedances have also been interpolated and visualized over time (Figure 7). In general, impedances varied individually and changed over time. Some subjects (such as EP04, EP10, EP11, and EP13) showed a stable pattern over time. In EP03, impedances remained relatively stable during the first 100 days of CI rehabilitation but then increased substantially. In subjects EP08 and EP14, impedances also increased but only for the most basal electrodes. Others preserved low impedances for a part of the array (EP12) or developed these (EP06).

### 3.6. Behavioral thresholds

Supplementary Figure 3 shows that median M and T levels increased over time during CI rehabilitation.

### 3.7. Exploratory *post-hoc* correlation analysis

**Figure 8:**
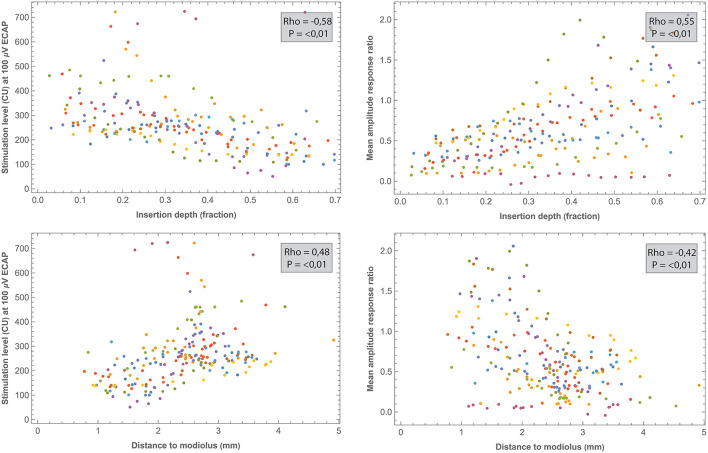
Comparisons between intraoperative ECAP parameters (100 μv ECAP and mean amplitude response ratio) and electrode positioning. All correlations were significant after the Bonferroni correction (*p* < 0.001). Each subject (contributing 16 electrodes if no missing values) is represented by a different color. Outliers of one NRI measurement (EP12) were out of the window and are not shown.

**Figure 9:**
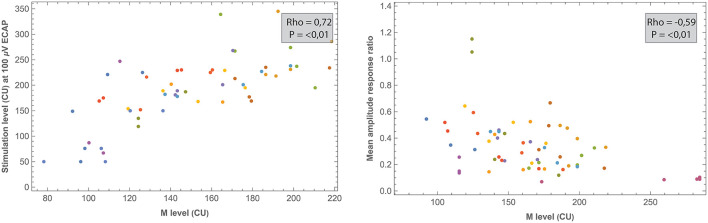
Comparisons between ECAP parameters and measured M levels. All measurements were recorded 1 week after CI activation. Each subject (contributing five data points if no missing values) is represented by a different color.

Correlations between electrode positioning and electrophysiological measures (during surgery and 1 week after CI activation) are presented in Table 3. After correction for multiple comparisons (*p*-value < 0.001), both intra- and postoperative 100 μV ECAP and mean amplitude response ratio significantly correlated with insertion depth (as a fraction relative to cochlear duct length) and distance to the modiolus, but not with distance to the MW. Specifically, 100 μV ECAP increased, and the mean amplitude response ratio decreased (thus both indicating less neural excitation for a similar level of stimulation) with insertion depth (Figure 8). The strength of correlations was weak to moderate (38). IQR amplitude response ratio significantly correlated with insertion depth (both intraoperative and postoperative) and with distance to the modiolus (postoperative). Intraoperatively, a significant correlation between impedances and electrode positioning was found, where higher impedances were found toward the apical portion of the cochlea and when located closer to the modiolus (Supplementary Figure 4). As shown in Table 4 and visualized in Figure 9, M levels were strongly correlated (non-linear) with 100 uV ECAP (*r*_s_ = 0.72) and moderately correlated with mean amplitude response ratio (*r*_s_ = −0.59).

**Table 3:** Spearman correlation analysis showing relationships between electrode positioning and ECAP measures, impedances, and behavioral stimulation levels.

			**Insertion depth**	**Distance to modiolus**	**Distance to MW**
**NRI**					
100 μv ECAP	Intraoperative	*r*_s_	−0.58	0.48	0.17
		*p*	< 0.01^**•^	< 0.01^**•^	0.01^*^
	Activation +1 week	*r*_s_	−0.33	0.42	0.15
		*p*	< 0.01^**•^	< 0.01^**•^	0.05^*^
Mean amplitude response ratio	Intraoperative	*r*_s_	0.55	−0.42	−0.05
		*p*	< 0.01^**•^	< 0.01^**•^	0.45
	Activation +1 week	*r*_s_	0.36	−0.40	−0.16
		*p*	< 0.01^**•^	< 0.01^**•^	0.02^*^
IQR amplitude response ratio	Intraoperative	*r*_s_	0.28	−0.07	0.13
		*p*	< 0.01^**•^	0.30	0.05^*^
	Activation +1 week	*r*_s_	0.36	−0.37	−0.15
		*p*	< 0.01^**•^	< 0.01^**•^	0.03^*^
**Impedances**					
Impedance	Intraoperative	*r*_s_	0.42	−0.21	−0.01
		*p*	< 0.01^**•^	< 0.01^**•^	0.90
	Activation +1 week	*r*_s_	−0.09	0.07	−0.05
		*p*	0.16	0.31	0.42
**Behavioral stimulation levels**					
T levels	Activation +1 week	*r*_s_	−0.28	0.22	0.25
		*p*	0.02*	0.07	0.04*
M levels	Activation +1 week	*r*_s_	−0.05	0.14	−0.01
		*p*	0.67	0.26	0.94

Time points were either intraoperative or 1 week after CI activation. Significant correlations (without Bonferroni correction) have been marked with asterisks (^*^p < 0.05, ^**^p < 0.01). Correlations that remained significant after Bonferroni correction (*p* < 0.001) have been marked with a filled circle at the end of double asterisks (^**•^).

**Table 4:** Spearman correlation analysis showing relationships between ECAP outcomes and both impedances and stimulation levels.

		**Impedances**	**T levels**	**M levels**
100 μv ECAP	*r*_s_	0.06	0.06	0.72
	*p*	0.42	0.69	< 0.01^**•^
Mean amplitude response ratio	*r*_s_	−0.12	−0.29	−0.59
	*p*	0.05^*^	0.02	< 0.01^**•^
IQR amplitude response ratio	*r*_s_	−0.20	−0.23	−0.41
	*p*	< 0.01^**•^	0.05	< 0.01^**•^

All measurements were recorded 1 week after CI activation. Significant correlations (without Bonferroni correction) have been marked with asterisks (^*^p < 0.05, ^**^p < 0.01). Correlations that remained significant after Bonferroni correction (*p* < 0.001) have been marked with a filled circle at the end of double asterisks (^**•^).

## 4. Discussion

### 4.1. Imaging and electrode positioning

Electrode positioning was determined by marking contacts in 3D space in relation to the nearest point on the LW. Previously, this method was applied in a case report study and has now been evaluated in a larger sample (32). The 3D reconstruction used in this method allows for a qualitative assessment that is independent of reconstruction planes, as is the case when evaluating electrode positioning in 2D (39). Using this method, results showed that study subjects had an average cochlear length (measured as the extent of the LW) of 38.48 mm, which is somewhat longer than the average lengths of 35.8 mm reported by Meng et al. (40) and 37.9 mm by Würfel et al. (41). However, it is known that there exists a large variation in individual cochlear length across subjects (41). The median insertion depth of the most apical electrode was 23.73 mm, or 412° when expressed as an angular component. Previous studies on the positioning of the Midscala electrode array reported similar angular insertion depths of 389° and 437° (42, 43).

Based on visual inspection, Figure 3 shows that electrodes were commonly positioned closer to the medial wall at the basal and apical portions of the array. This is partially in line with Van der Jagt et al. (42), who reported this observation only for apical electrodes. Electrode-to-modiolus distance in the current study was highest for basal contacts and decreased gradually toward the apex. This pattern is consistent with results from previous studies examining electrode arrays with a precurved design (9, 28). Davis et al. (44) examined the modiolar proximity of the Midscala electrode but localized the complete modiolus instead of only the midmodiolar axis, which complicates direct comparisons. By defining the modiolus as an axis, the basal part of the cochlea is likely to be located further away from the axis, because the modiolus is wider basally than more apically. It is currently unknown which method has the best clinical value as the benefits or trade-offs of electrode positioning are not clear.

In a recent meta-analysis, it has been reported that for perimodiolar electrode arrays, such as the Midscala, translocation to scala vestibuli occurs in 43% of patients (45). An *in vivo* study examining the scalar position of the Midscala confirms this observation (46). Similarly, in the current study, translocation occurred in 50% of subjects. In one subject, a scala vestibuli insertion occurred. Loss of residual hearing due to surgery was 12 and 27 dB HL in subjects with and without a scalar translocation, respectively. Preoperative residual hearing however was already lower in subjects that eventually had a translocation. According to the literature, most arrays translocate around 180° depth, which is around the first ascending turn of the cochlea. In the current study, translocation most often occurred at electrodes 10 and 11, which were located within the range of 115–136°. This difference might be explained by variations in cochlear morphology and surgical approach. Also, methods to identify scalar location varied between studies. Some researchers used image-processing algorithms to identify scalar location while others relied on visual inspection.

### 4.2. Electrically evoked compound action potential

In the current study, three outcome measures were derived from intra- and postoperative ECAP growth functions to analyze neural response: 100 μV ECAP, mean amplitude response ratio, and IQR amplitude response ratio. These outcome measures were significantly correlated to certain aspects of electrode positioning. Specifically, 100 μV ECAP increased and both the mean and IQR amplitude response ratios decreased with increasing distance to the modiolus. In other words, electrodes that were located further from the midmodiolar axis generally required more current to elicit the same ECAP response. In contrast, no significant correlation after the correction was found between ECAP measures and electrode distance to the MW. This is consistent with a study from Schvartz-Leyzac et al. (9), who hypothesized that the site of excitation for residual spiral ganglion cells occurs more centrally in the modiolus and is not restricted to the more peripheral portion of neurons adjacent to the MW.

Our results also show that 100 μV ECAP increased and both mean and IQR amplitude response ratio decreased with increasing insertion depth. Thus, electrodes located toward the apical part of the cochlea induced greater ECAP responses compared to basal electrodes. Correlations were significant for both intra- and postoperative measurements, but relationships were stronger for intraoperative measurements. Although measured with different ECAP outcomes, these results are in line with previous reports showing higher ECAP amplitudes and slopes for electrodes located in the apical region of the cochlea (19–21). This can be partially explained by the observed higher survival of auditory neurons toward the apex (47). Also, apical electrodes are likely to be located closer to the modiolar neurons due to the smaller diameter of the cochlear apex.

When comparing intraoperative and postoperative measurements, 100 μV ECAP responses were similar except for electrodes located toward the basal portion of the cochlea that needed higher stimulation levels during surgery (Figure 4). Other studies reported higher ECAP thresholds intraoperatively compared to postoperative measurements for all electrodes. This is most likely related to physiological changes between surgery and fitting, induced by electrical stimulation, as also indicated by impedance measurements (48, 49). Also, anesthesia during surgery has been reported to impact ECAP response (50). In those subjects with a scalar translocation, otopathological changes such as fibrosis formation and neural degeneration might have occurred due to cochlear damage (51). As part of the standard clinical routine, CI settings between subjects also differed in terms of fitted M and T levels, which exposed subjects to different levels of electrical stimulation in day-to-day living (50). Furthermore, it should be noted that differences between intra- and postoperative measurements might have been affected by measurement ranges. For example, during surgery, ECAP was measured at relatively high stimulation levels, while postoperative measurements were limited to subjects' comfortable levels (refer to Figure 5). This discrepancy in stimulation range limits the range of the ECAP growth functions and as such reduces the precision and affects the values of the derived parameters during normal clinical follow-up visits. Smaller increments and extending the measurement range by lowering the stimulation could possibly be useful in the future to increase the amount of sampled data. There was a greater variation in 100 μV ECAP for the intraoperative results, which might relate to bigger step sizes in stimulation level when compared to postoperative measurements. The mean amplitude response ratio was different between intraoperative and postoperative measurements with an increased difference toward the basal electrodes. IQR amplitude response ratio did not show this pattern with the exception of higher intraoperative values at electrodes 6–11. In general, median ECAP measures at 1, 8, and 26 weeks postactivation remained stable over time, which is consistent with current literature (48, 52). On an individual level, however, variation over time existed, which might also be explained by the factors mentioned earlier.

Typically, researchers have used ECAP thresholds, slopes, or amplitudes at specific stimulation levels to analyze ECAP. Thresholds (defined as the minimum amount of current needed to elicit a measurable response) are often estimated by linear extrapolation of the ECAP growth function. However, ECAP growth is often not linear (53), as is also illustrated in this study (Supplementary Figure 2). As an alternative, in the current study, the amount of stimulation needed to elicit an ECAP response of 100 μV was used as an outcome. This arbitrary choice was motivated by the estimate that this level should be well-above the noise floor of ECAP recordings, and below the maximum ECAP amplitude (19). However, depending on the location, the 100 μV level was not reached in a considerable number of patients. This might be explained by variance in maximum stimulation level between visits or actual differences in neural excitation. Nevertheless, these occurrences have been recorded as missing, and thus might have influenced results since actual 100 μV ECAP levels might have been detected if subjects were stimulated at higher levels. As a different outcome, the slope of the linear growth function has often been used to characterize the rate of growth as a function of stimulation level. In this study, we proposed a new method by calculating ratios between stimulation level and ECAP response across the entire input–output function. Compared to the traditional slope based on linear regression, we believe calculating the mean or interquartile range of these ratios might be a suitable alternative since individual ECAP growth functions are almost never linear, as evidently shown here. Also, this parameter is directly derived from available data. In future research, the predictive value of different ECAP derivatives should be addressed and compared to neural health measures.

### 4.3. Impedances

A significant, but weak to moderate, correlation was found for intraoperative impedances and both insertion depth and electrode-to-modiolus distance, with higher impedances for electrodes located toward the apical portion of the cochlea and for electrodes with decreased distance to the modiolus. Postoperative impedances were not significantly related to electrode positioning. Previous studies have published mixed results regarding the link between impedances and electrode positioning. For example, some studies reported higher impedances at the apex of the cochlea compared to the base (25, 26, 28), while Saunders et al. (27) reported an inverse relationship. Studies that linked impedance measurements to electrode-to-modiolus distance did not find a clear relationship (22, 27, 28). Over time, median impedances were low during surgery, increased significantly when measured after CI activation, and then remained stable for the first half year of CI rehabilitation. This pattern was not evident for every subject, demonstrating variability in impedances over time among cochleas. Previous studies have also reported a large increase in impedance values after surgery (54–56). This might be explained by fibrous tissue growth following surgery (57). Interestingly, ECAP measures did not significantly correlate (after Bonferroni correction) with impedances.

### 4.4. Behavioral thresholds

After correction for multiple comparisons, no significant correlation was found between electrode positioning and behavioral thresholds. In a previous study, higher fitting levels were found toward the basal end of the cochlea (58). Here, the electrode-to-modiolus distance was weakly correlated with stimulation level, as was also the case in the current study. Over time, both T and M levels increased during CI rehabilitation, as can be expected based on clinical practice and previous research (59).

Previously, studies including devices from multiple manufacturers have reported only a moderate correlation between ECAP and behavioral stimulation levels (11, 23). In the current prospective study, M levels strongly correlated with 100 μV ECAP (*r*_s_ = 0.72) and moderately with both mean (*r*_s_ = −0.59) and IQR (*r*_s_ = −0.41) amplitude response ratio. Further research might address whether these ECAP parameters have clinical value in predicting or fitting behavioral thresholds. No significant relationship was found between ECAP parameters and T levels.

### 4.5. Limitations

The sample size in this prospective study is limited. Relationships between outcomes have therefore only been tested with exploratory correlation analysis. Ideally, in a larger study group, additional statistics would have been performed to identify shared variance between factors. Also, imaging procedures and electrode localization did not come without limitations. Specifically, the identification of the medial wall was complicated due to limited image resolution. This drives the prerequisite of MRI administration instead of CT. Also, the modiolus was localized as a midmodiolar axis while in reality, it is a three-dimensional curved structure. Possibly, migration of electrodes might have affected results although most arrays achieve a stable position, as studied before (60).

An important limitation of the ECAP results presented in this study relates to the diversity in measurement methods. The maximum stimulation level was not fixed but limited according to the subject's comfort or clinical practice (intraoperatively). Smaller increments in stimulation level, especially around the stimulation-reaction threshold might give more information on the sensitivity of the remaining auditory nerves. Also, a baseline measurement at 0 CU was not included to establish the noise floor. Identification of P2 and N1 peaks was performed automatically by fitting software Soundwave but was manually changed if subjectively judged necessary.

## 5. Conclusion

In this prospective study, electrode positioning in CI subjects was determined with detailed 3D imaging and related to objective CI measures (e.g., impedances, behavioral thresholds, and ECAPs, which were characterized using multiple parameters). By measuring multiple ECAP and impedance measurements over time, and the corresponding visualizations, which we have shown for the first time, we hope to provide a better estimate of how these measures develop over time and explore factors that explain variability. On a group level, ECAPs and impedances showed consistent trends over time, but high variability existed among subjects and between different positions in the cochlea. Specifically, electrodes that were located closer to the apex of the cochlea and closer to the modiolus showed higher neural excitation (represented by ECAP parameters) and higher impedances. Only a weak correlation was found between ECAP and impedances. In some patients, major changes in ECAP and impedances occurred over time. This might have been related to scala translocations of the electrode array, which occurred in half of the study subjects, and potentially induced cochlear trauma. Maximum loudness comfort levels were correlated strongly with the level of current needed to elicit a response of 100 μV ECAP. Further research might address whether this ECAP parameter or the newly introduced other parameters and visualizations, have clinical value in fitting electrode contacts or can be used as a measure of neural excitability to be linked to intracochlear trauma or surviving auditory neurons.

## Data availability statement

The raw data supporting the conclusions of this article will be made available by the authors, without undue reservation.

## Ethics statement

The studies involving human participants were reviewed and approved by Ethics Committee of the Maastricht University Medical Center. The patients/participants provided their written informed consent to participate in this study.

## Author contributions

LL and JD performed subject measurements and organized the database. LL and MH performed the statistical analysis. The first draft of the manuscript was written by LL. All authors contributed to conception and design of the study, manuscript revision, read, and approved the submitted version.

## Supplementary material

Supplementary Figure 1 Median pure-tone air conduction thresholds in the CI ear before surgery and after surgery (measured at first fitting). If no response could be recorded within the limits of the audiometer, a value of 5 dB HL greater than the maximum tested level was attributed. Bands indicate the first and third quartiles.

Supplementary Figure 2 Contour plots for all subjects showing ECAP responses (μV) in relation to stimulation level (CU) across the electrode array. Colors indicate ECAP values according to the legend. Measurements have been performed during surgery, and three times during the first 6 months of CI rehabilitation.

Supplementary Figure 3 Median measured M and T levels (lower values) across the electrode array obtained during three visits within the first 6 months of CI rehabilitation. Measurements were performed on stimulation channels (electrode pairs) 1–2, 4–5, 7–8, 11–12, and 15–16 and interpolation was applied in between. Bands indicate the first and third quartiles.

Supplementary Figure 4 Comparisons between intraoperative impedances and electrode positioning. Each subject (contributing 16 electrodes if no missing values) is represented by a different color. Impedance values were automatically rounded to the nearest hundred by Soundwave software.

Supplementary Table 1 Electrode positioning across the electrode array. IQR, interquartile range.
