# Supplementary figures and images for: Toward neural health measurements for cochlear implantation: The relationship among electrode positioning, the electrically evoked action potential, impedances and behavioral stimulation levels
Source: Front Neurol. 2023 Feb 9;14:1093265. doi: 10.3389/fneur.2023.1093265 (PMC9948626; doi:10.3389/fneur.2023.1093265)

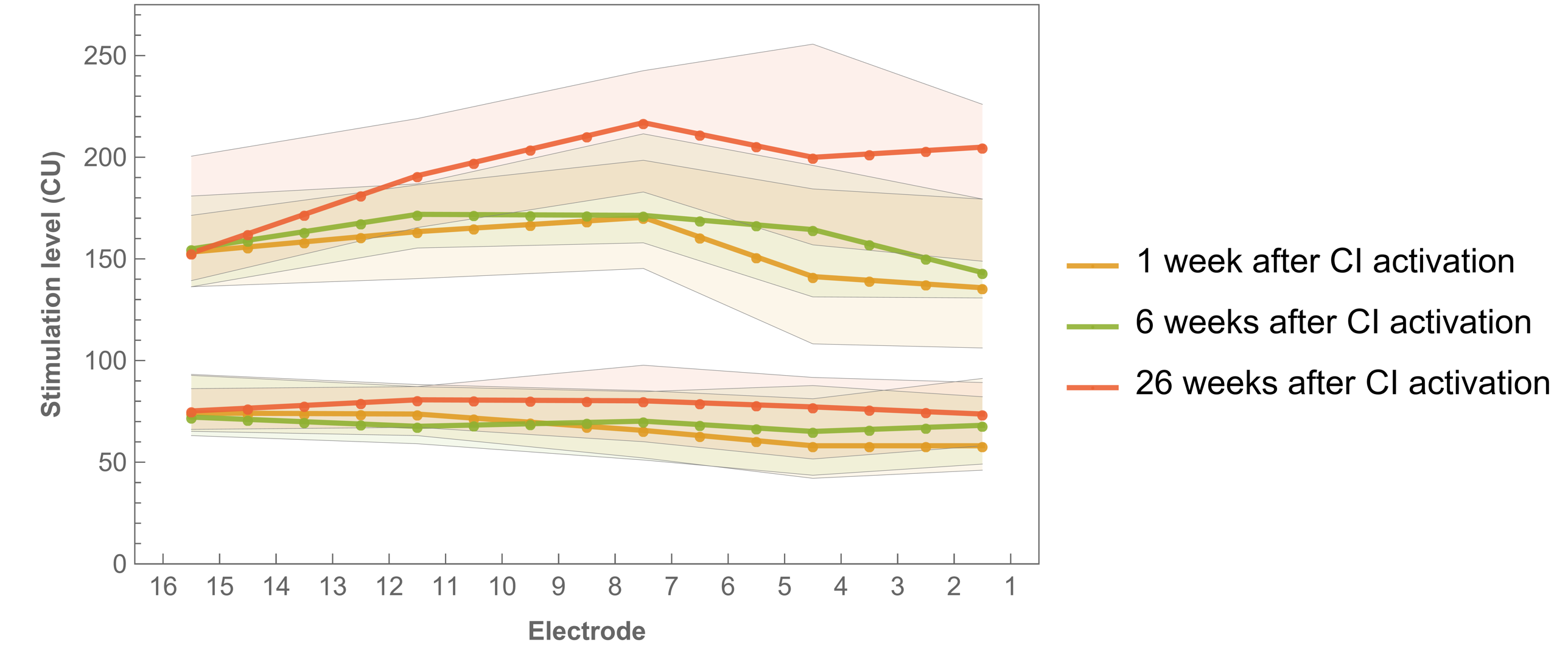

Supplement: Supplementary Figure 3 — Median measured M and T levels (lower values) across the electrode array obtained during three visits within the first 6 months of CI rehabilitation. Measurements were performed on stimulation channels (electrode pairs) 1–2, 4–5, 7–8, 11–12, and 15–16 and interpolation was applied in between. Bands indicate the first and third quartiles. [file Image_3.TIF]

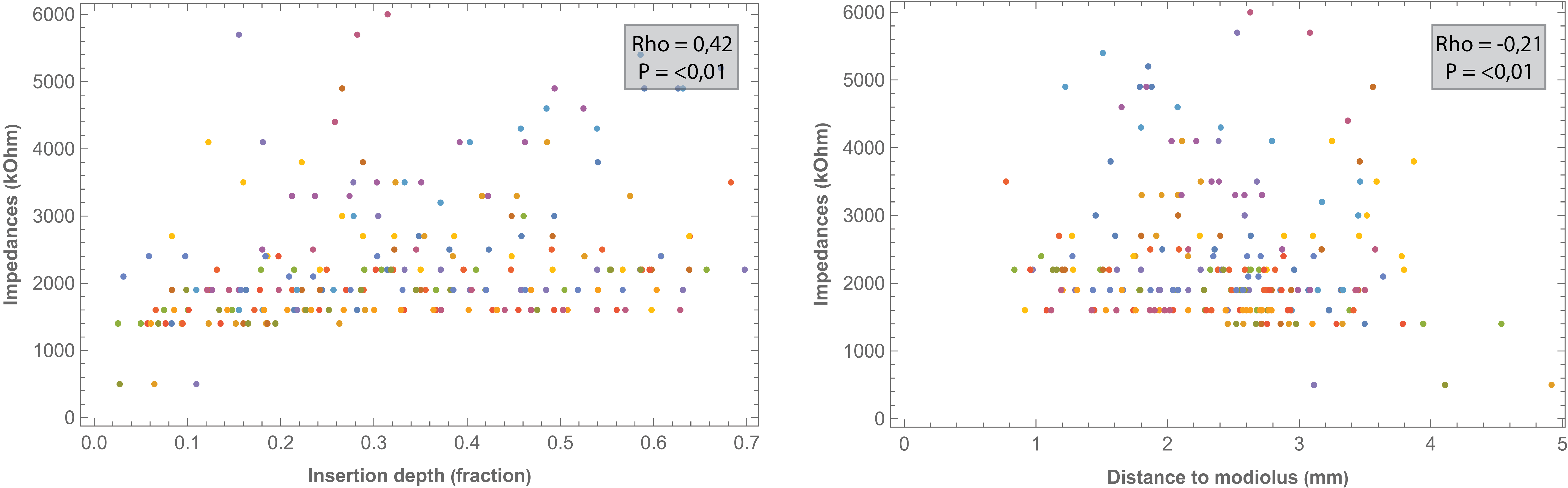

Supplement: Supplementary Figure 4 — Comparisons between intraoperative impedances and electrode positioning. Each subject (contributing 16 electrodes if no missing values) is represented by a different color. Impedance values were automatically rounded to the nearest hundred by Soundwave software. [file Image_4.TIF]
