# Supplementary material for: Toward neural health measurements for cochlear implantation: The relationship among electrode positioning, the electrically evoked action potential, impedances and behavioral stimulation levels
Source: Front Neurol. 2023 Feb 9;14:1093265. doi: 10.3389/fneur.2023.1093265 (PMC9948626; doi:10.3389/fneur.2023.1093265)
Supplement: Supplementary Table 1 — Electrode positioning across the electrode array. IQR, interquartile range. [file Table_1.docx]

|  |  | EL16 | EL15 | EL14 | EL13 | EL12 | EL11 | EL10 | EL9 | EL8 | EL7 | EL6 | EL5 | EL4 | EL3 | EL2 | EL1 |
| --- | --- | --- | --- | --- | --- | --- | --- | --- | --- | --- | --- | --- | --- | --- | --- | --- | --- |
| Insertion depth (mm) | Median | 2.52 | 3.71 | 5.38 | 6.97 | 7.94 | 9.04 | 10.23 | 11.41 | 12.48 | 13.84 | 15.38 | 17.08 | 18.58 | 20.06 | 22.08 | 23.73 |
|  | IQR | 1.95 | 2.75 | 2.85 | 2.84 | 2.48 | 2.36 | 2.19 | 2.24 | 2.58 | 2.13 | 2 | 2.35 | 2.73 | 3.22 | 2.65 | 2.72 |
| Angular insertion depth (◦) | Median | 28 | 42 | 63 | 83 | 98 | 115 | 136 | 152 | 172 | 192 | 220 | 253 | 290 | 327 | 362 | 412 |
|  | IQR | 23 | 29 | 33 | 31 | 36 | 32 | 21 | 26 | 28 | 28 | 34 | 28 | 45 | 60 | 43 | 47 |
| Tonotopic location (Hz) | Median | 14394 | 12195 | 9882 | 8408 | 7131 | 6206 | 5284 | 4499 | 3846 | 3187 | 2677 | 2135 | 1685 | 1341 | 1100 | 852 |
|  | IQR | 3534 | 4533 | 4060 | 2717 | 2290 | 2152 | 1705 | 1000 | 1244 | 865 | 964 | 644 | 690 | 561 | 379 | 319 |
| Distance to the medial wall (mm) | Median | 0.86 | 0.66 | 0.76 | 0.86 | 0.99 | 1.04 | 0.97 | 1.00 | 1.00 | 0.93 | 0.83 | 0.80 | 0.71 | 0.71 | 0.60 | 0.59 |
|  | IQR | 0.49 | 0.47 | 0.49 | 0.40 | 0.39 | 0.58 | 0.48 | 0.45 | 0.40 | 0.35 | 0.36 | 0.39 | 0.32 | 0.17 | 0.24 | 0.19 |
| Distance to the lateral wall (mm) | Median | 1.52 | 1.49 | 1.46 | 1.29 | 1.01 | 0.87 | 0.92 | 0.89 | 0.95 | 1.07 | 1.05 | 1.24 | 1.26 | 1.21 | 1.21 | 1.06 |
|  | IQR | 0.75 | 0.58 | 0.49 | 0.55 | 0.69 | 0.68 | 0.42 | 0.29 | 0.58 | 0.60 | 0.44 | 0.40 | 0.22 | 0.50 | 0.80 | 0.72 |
| Distance to the modiolus (mm) | Median | 3.43 | 3.05 | 2.79 | 2.81 | 2.70 | 2.75 | 2.71 | 2.63 | 2.51 | 2.29 | 2.14 | 1.90 | 1.79 | 1.53 | 1.24 | 1.20 |
|  | IQR | 0.68 | 0.38 | 0.76 | 0.37 | 0.56 | 0.61 | 0.35 | 0.41 | 0.48 | 0.47 | 0.39 | 0.32 | 0.15 | 0.13 | 0.27 | 0.30 |

**Supplemental Table 1.** Electrode positioning across the electrode array. IQR = Interquartile Range.
